# Identification of Prognostic Biomarker Signatures and Candidate Drugs in Colorectal Cancer: Insights from Systems Biology Analysis

**DOI:** 10.3390/medicina55010020

**Published:** 2019-01-17

**Authors:** Md. Rezanur Rahman, Tania Islam, Esra Gov, Beste Turanli, Gizem Gulfidan, Md. Shahjaman, Nilufa Akhter Banu, Md. Nurul Haque Mollah, Kazim Yalcin Arga, Mohammad Ali Moni

**Affiliations:** 1 Department of Biotechnology and Genetic Engineering, Islamic University, Kushtia-7003, Bangladesh; taniaislam1304@gmail.com (T.I.); nab_bt_iu@yahoo.com (N.A.B.); 2 Department of Biochemistry and Biotechnology, School of Biomedical Science, Khwaja Yunus Ali University, Sirajgonj-6751, Bangladesh; 3 Department of Bioengineering, Adana Science and Technology University, Adana-01250, Turkey; egov@adanabtu.edu.tr; 4 Department of Bioengineering, Marmara University, Istanbul-34722, Turkey; bcalimlioglu@gmail.com (B.T.); gizemgulfidn@gmail.com (G.G.); kazim.arga@marmara.edu.tr (K.Y.A.); 5 Department of Bioengineering, Istanbul Medeniyet University, Istanbul-34700, Turkey; 6 Department of Statistics, Begum Rokeya University, Rangpur-5400, Bangladesh; shahjaman_brur@yahoo.com; 7 Laboratory of Bioinformatics, Department of Statistics, University of Rajshahi, Rajshahi-6205, Bangladesh; mollah.stat.bio@ru.ac.bd; 8 The University of Sydney, Faculty of Medicine and Health, Sydney Medical School, Discipline of Biomedical Science, Sydney, NSW 2006, Australia

**Keywords:** colorectal cancer, differentially expressed genes, biomarkers, protein–protein interaction, reporter biomolecules, candidate drugs, systems biology, drug repositioning

## Abstract

*Background and objectives*: Colorectal cancer (CRC) is the second most common cause of cancer-related death in the world, but early diagnosis ameliorates the survival of CRC. This report aimed to identify molecular biomarker signatures in CRC. *Materials and Methods*: We analyzed two microarray datasets (GSE35279 and GSE21815) from the Gene Expression Omnibus (GEO) to identify mutual differentially expressed genes (DEGs). We integrated DEGs with protein–protein interaction and transcriptional/post-transcriptional regulatory networks to identify reporter signaling and regulatory molecules; utilized functional overrepresentation and pathway enrichment analyses to elucidate their roles in biological processes and molecular pathways; performed survival analyses to evaluate their prognostic performance; and applied drug repositioning analyses through Connectivity Map (CMap) and geneXpharma tools to hypothesize possible drug candidates targeting reporter molecules. *Results*: A total of 727 upregulated and 99 downregulated DEGs were detected. The PI3K/Akt signaling, Wnt signaling, extracellular matrix (ECM) interaction, and cell cycle were identified as significantly enriched pathways. Ten hub proteins (ADNP, CCND1, CD44, CDK4, CEBPB, CENPA, CENPH, CENPN, MYC, and RFC2), 10 transcription factors (ETS1, ESR1, GATA1, GATA2, GATA3, AR, YBX1, FOXP3, E2F4, and PRDM14) and two microRNAs (miRNAs) (miR-193b-3p and miR-615-3p) were detected as reporter molecules. The survival analyses through Kaplan–Meier curves indicated remarkable performance of reporter molecules in the estimation of survival probability in CRC patients. In addition, several drug candidates including anti-neoplastic and immunomodulating agents were repositioned. *Conclusions*: This study presents biomarker signatures at protein and RNA levels with prognostic capability in CRC. We think that the molecular signatures and candidate drugs presented in this study might be useful in future studies indenting the development of accurate diagnostic and/or prognostic biomarker screens and efficient therapeutic strategies in CRC.

## 1. Introduction

Colorectal cancer (CRC) is the second most common cause of mortality of men and women in the world [1]. The number of CRC cases is still increasing, and the global burden of CRC is expected to increase by 60% to more than 2.2 million new cases and 1.1 million deaths by 2030 [2]. Like other cancers, a number of factors such as genetic factors, epigenetic alterations, diet, and environmental factors contribute to the progression and metastasis of CRC [3,4]. Despite the comprehensive studies (as reviewed by Reference [5]), the molecular mechanisms of CRC pathogenesis are only partially understood. Several biomarkers (KRAS and BRAF) are used to detect CRC, but these biomarkers are not sufficiently sensitive and specific; consequently, there is an urgent need for the identification of efficacious biomarkers, therapeutic targets, and agents for early diagnosis, prevention, and personalized therapy in CRC [6].

Gene expression profiling technologies were employed for years to identify genetic alterations at the transcriptional level that pave the way to candidate biomarkers in human diseases including cancers [7,8,9]. These biomarkers may be used in early detection and/or serve as novel therapeutic targets. Hundreds of differentially expressed genes (DEGs) were identified in CRC from microarray data [10,11]; however, their roles within human signaling networks and their transcriptional regulatory mechanisms via transcription factors (TFs) and microRNAs (miRNAs) were not studied in detail within a network biomedicine approach. The regulatory biomolecules might be attractive biomarkers since several reports proposed microRNAs (miRNAs) that act as key players in CRC as prognostic biomarkers [12,13].

**Figure 1:**
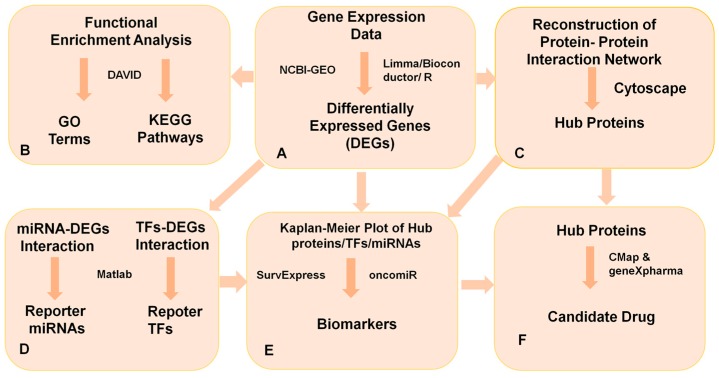
The integrative analytical pipeline employed in the present study. (**A**) The colorectal cancer (CRC) datasets were analyzed under the Bioconductor platform in R. We used linear models for microarray data (LIMMA) to detect the differentially expressed genes (DEGs) in CRC compared to normal samples. (**B**) Gene ontology (GO) terms and molecular pathways were identified by DEGs enrichment via the Database for Annotation, Visualization and Integrated Discovery (DAVID). (**C**) The hub proteins were identified by protein–protein interaction (PPI) analysis. (**D**) The reporter feature algorithm was used to identify reporter biomolecules as transcriptional regulatory elements. (**E**) The survival analysis of the hub biomolecules was done through The Cancer Genome Atlas (TCGA) CRC datasets via SurvExpress and oncomiR. (**F**) The candidate drug molecules were identified by Connectivity Map (cMap) and geneXpharma.

The power of multi-omics analyses within the network biomedicine perspective [14] in the elucidation of molecular signatures in human diseases was previously shown in many human diseases such as head and neck cancers [15], esophageal squamous cell carcinoma [16], triple negative breast cancer [17], cervical cancer [18], ovarian cancer [19] and ovarian diseases [20], psoriasis [21], and type 2 diabetes [22]. Therefore, in this study, systems-based approaches were considered to explore the potential biomarker signatures at protein (i.e., hub proteins and transcription factors (TFs)) and RNA levels (i.e., miRNAs and messenger RNAs (mRNAs)) (Figure 1). For this purpose, we considered mutual DEGs identified from two independent gene expression profiling studies to maintain robustness, integrated this information with human biomolecular networks (i.e., protein–protein interaction and transcriptional/post-transcriptional regulatory networks) to identify reporter signaling and regulatory molecules, utilized functional overrepresentation and pathway enrichment analyses to elucidate the roles of reporter molecules in biological processes and molecular pathways, and performed survival analyses to evaluate their prognostic performance as potential biomarkers in CRC. In addition, several candidate drugs were repositioned in CRC using in silico drug repositioning tools, Connectivity Map (CMap) [23] and geneXpharma [24], considering these biomarker signatures as therapeutic targets.

## 2. Materials and Methods

### 2.1. High-Throughput Microarray Gene Expression Datasets

To analyze mRNA signatures in CRC samples compared to normal tissues, two gene expression datasets obtained using Agilent microarrays in independent experiments, GSE35279 [25] and GSE21815 [26], were downloaded from the Gene Expression Omnibus (GEO) database [27], which is a public functional genomics data repository supporting MIAME compliant data submissions. Consequently, a total of 220 specimens (206 CRC specimens and 14 normal samples) were comparatively analyzed.

### 2.2. Identification of Differentially Expressed Genes

To characterize differentially expressed genes (DEGs), each dataset was normalized by means of the robust multi-array average (RMA) expression measure [28], and DEGs were identified from the normalized log-expression values using the multiple testing option of LIMMA (linear models for microarray data) [29] using the R/Bioconductor platform (version R × 64 3.4.1). Benjamini–Hochberg’s method was used to control the false discovery rate. An adjusted *p*-value threshold of 0.01 with a fold-change cutoff of 2 was used to determine the statistical significance of differential expression.

### 2.3. Gene Ontology and Pathway Analysis

Clustering of DEGs and reporter molecules into functional groups (i.e., biological processes and molecular pathways) was performed via DAVID’s functional annotation tool [30]. In the analyses, the Kyoto Encyclopedia of Genes and Genomes (KEGG) [31] was preferably used as the pathway database and the Gene Ontology (GO) project [32] was used as the annotation source for biological processes and molecular functions. Fisher’s exact test was used to evaluate the statistical significance. The *p*-values were corrected via Benjamini–Hochberg’s method, and an adjusted *p*-value threshold (adj-*p* < 0.05) was used for all enrichment analyses.

### 2.4. Reconstruction and Analysis of Protein–Protein Interaction (PPI) Network in CRC

We recruited the previously reconstructed high-confidence PPI network of *Homo sapiens* [33] consisting of 288,033 physical interactions between 21,052 proteins to construct a PPI subnetwork around the proteins encoded by the identified DEGs. The subnetwork was visualized and analyzed via Cytoscape (v3.4 and 2.8.3) [34]. The topological analysis was performed to characterize the network properties through the Cyto-Hubba plugin [35]. The dual-metric approach [17,22] utilizing a local (i.e., degree) and a global (i.e., betweenness centrality) metric was simultaneously employed to define hub proteins. The modules in the PPI sub-networks were identified using MCODE plug-in [36] in Cytoscape. The modules were further analyzed through enrichment analyses in DAVID’s functional annotation tool [30].

### 2.5. Identification of Reporter Biomolecules

To identify reporter regulatory molecules (i.e., TFs and miRNAs) around which significant changes occur at the transcriptional level, we employed the comprehensive human transcriptional and post-transcriptional regulatory network [37], consisting of the experimentally verified TF–target gene and miRNA–target gene interactions from HTRIdb [38] and miRTarbase (Release 6.0) [39] databases. The reporter features algorithm [40] was used and implemented as described previously [15,18,20] to obtain *z*-scores and corresponding *p*-values of the molecules. The *p*-values were corrected via Benjamini–Hochberg’s method, and statistically significant (adj-*p* < 0.01) results were considered as reporter biomolecules.

### 2.6. Evaluation of the Prognostic Performance of Reporter Molecules

The prognostic power of reporter biomolecules (i.e., hubs, TFs, and miRNAs) was analyzed via multivariate Cox regression analysis as implemented in SurvExpress [41] and OncomiR [42], using independent gene expression (RNA sequencing (RNA-Seq) or miRNA-Seq) datasets obtained from The Cancer Genome Atlas (TGCA). The RNA-Seq dataset consisted of 467 samples with their clinical information, whereas the miRNA-Seq data included 424 patients. The patients were partitioned into low- and high-risk groups according to their prognostic index determined by SurvExpress or OncomiR. The differences in gene expression levels between the risk groups were represented via box-plots, and the statistical significance of the differences was estimated by Student’s *t*-test. The survival signatures of reporter biomolecules were evaluated by Kaplan–Meier plots, and a log-rank *p*-value < 0.05 was considered as the cut-off to describe statistical significance in all survival analyses.

### 2.7. Identification of Candidate Drugs

We simultaneously used the Connectivity Map (CMap) database [23] and geneXpharma tool [24] to identify potential candidate drugs. CMap stores the expression profiles from cultured human cells exposed to various small molecular agents. A total of 50,304 gene–drug interactions comprising 4344 genes and 11,939 drugs are presented in geneXpharma. The hypergeometric probability test was used to statistically associate drugs to CRC.

## 3. Results

### 3.1. Identification of Differentially Expressed Genes

**Figure 2:**
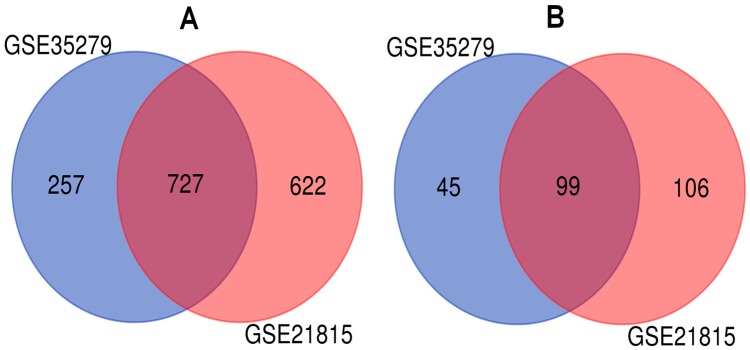
Identification of differentially expressed genes (DEGs) in colorectal cancer (CRC) from microarray CRC datasets: (**A**) the upregulated genes in the CRC expression profiling datasets; (**B**) the downregulated genes in the CRC expression profiling datasets.

**Figure 3:**
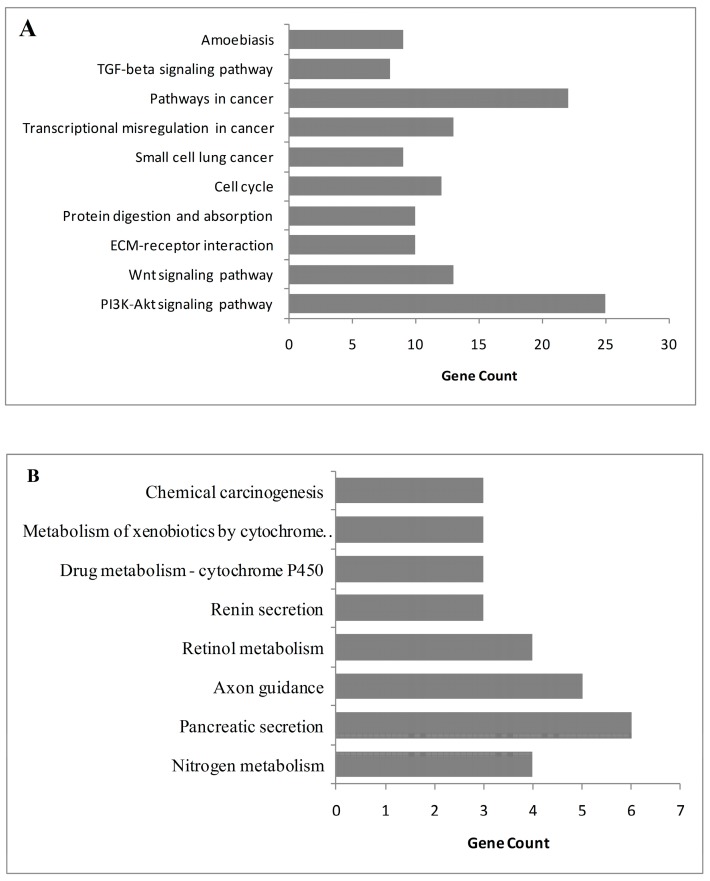
The significant pathways altered in colorectal cancer: (**A**) upregulated pathways in colorectal cancer; (**B**) downregulated pathways in colorectal cancer.

We studied two microarray CRC datasets (GSE35279 and GSE21815) from independent experiments to detect DEGs dysregulated in CRC samples compared to normal tissues. The analyses presented 727 upregulated and 99 downregulated genes mutually differentiated in both CRC datasets (Figure 2). Then, we performed gene set overrepresentation analyses to obtain the GO annotations (in terms of molecular function, biological process, and cellular component) and KEGG pathways significantly associated with DEGs. The top 5 GO terms for upregulated and downregulated DEGs are summarized in Table 1, and the significant molecular pathways altered in CRC are shown in Figure 3.

The overrepresentation analyses indicated the upregulation of collagen-associated processes, extracellular matrix (ECM) organization, and male gonad development. The upregulated proteins mainly had protein-binding activities and localized in extracellular environments or the cytoplasm. On the other hand, transport process, most specifically bicarbonate and chloride transport, were downregulated in CRC. Downregulated proteins mostly involved zinc ion binding, and hormone and chloride channel activities and were localized in the integral component of the plasma membrane (Table 1). In parallel to GO enrichment results, the PI3K/Akt signaling pathway, Wnt signaling pathway, cell cycle, lung cancer, ECM–receptor interaction, protein digestion and absorption, pathways in cancer, and TGF-β signaling pathway were upregulated in CRC (Figure 3A). Contrarily, nitrogen metabolism, pancreatic secretion, axon guidance, retinol metabolism, renin secretion, and chemical carcinogenesis pathways were downregulated in CRC (Figure 3B).

### 3.2. Analysis of Protein–Protein Interaction Network to Identify Hub Proteins

To identify hub proteins, a PPI sub-network around proteins encoded by the DEGs was constructed, and its topological analysis was performed. Following the scale-free degree distribution and small-world properties of biological networks, the presence of 10 hub proteins (ADNP, CCND1, CD44, CDK4, CEBPB, CENPA, CENPH, CENPN, MYC, and RFC2) was detected using degree and betweenness centrality metrics. These hub proteins may play significant key roles in signal transduction during the progression of CRC (Table 2). Two functional modules were revealed from the PPI network: module 1, consisting of IPO5, RBP2, and RAN, was associated with intracellular protein transport, and module 2, consisting of CENPN, CENPA, and CENPH, was enriched with sister chromatid cohesion, and kinetochore and nucleosome assembly (data not shown).

### 3.3. Identification of Regulatory Biomolecules

To identify reporter regulatory molecules (i.e., TFs and miRNAs) around which significant changes occur at transcriptional level, we integrated DEGs with a human transcriptional and post-transcriptional regulatory network and employed the adopted version of reporter features algorithm [20,40] for each dataset. Considering a statistical significance level of adj-*p* < 0.01, we identified 10 TFs (ETS1, ESR1, GATA1, GATA2, GATA3, AR, YBX1, FOXP3, E2F4, and PRDM14) and 10 miRNAs (miR-16-5p, miR-26b-5p, miR-124-3p, let-7b-5p, miR-92a-3p, miR-192-5p, miR-155-5p, miR-93-5p, miR-193b-3p, and miR-17-5p) as the mutual transcriptional regulatory components in both CRC datasets (Table 3).

### 3.4. Survival Analysis of Biomolecules

We performed the survival analysis of biomolecules (i.e., 10 hubs, 10 TFs, and 10 miRNAs) using CRC datasets from TCGA. Based on expression levels of reporter biomolecules and estimated survival probabilities, the patients were partitioned into two groups (i.e., high-risk and low-risk groups). The differential gene expression levels in high- and low-risk groups were represented by the box-plots and the estimated the survival probabilities were represented by Kaplan–Meier plots. In simulations, hub proteins, reporter TFs, and reporter miRNAs were considered as separate biomarker sets.

**Figure 4:**
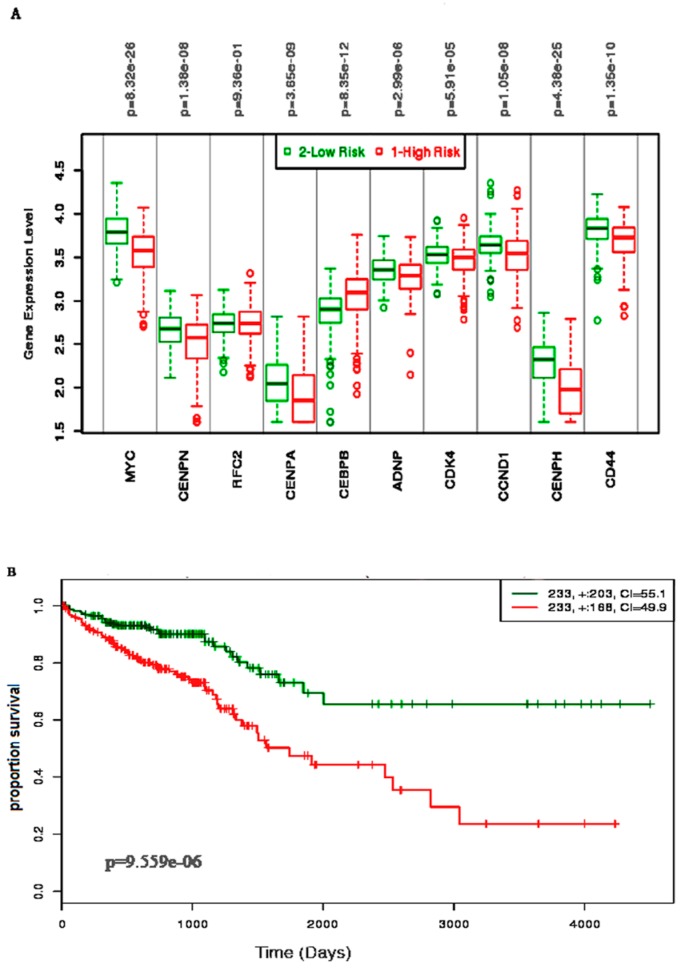
The survival analysis of the hub genes in the prognosis of colorectal cancer. (**A**) The box-plot represents the differential expression of the 10 hub genes in two risks groups. (**B**) The Kaplan–Meier plot represents the prognostic ability of the hub gene signatures in CRC.

**Figure 5:**
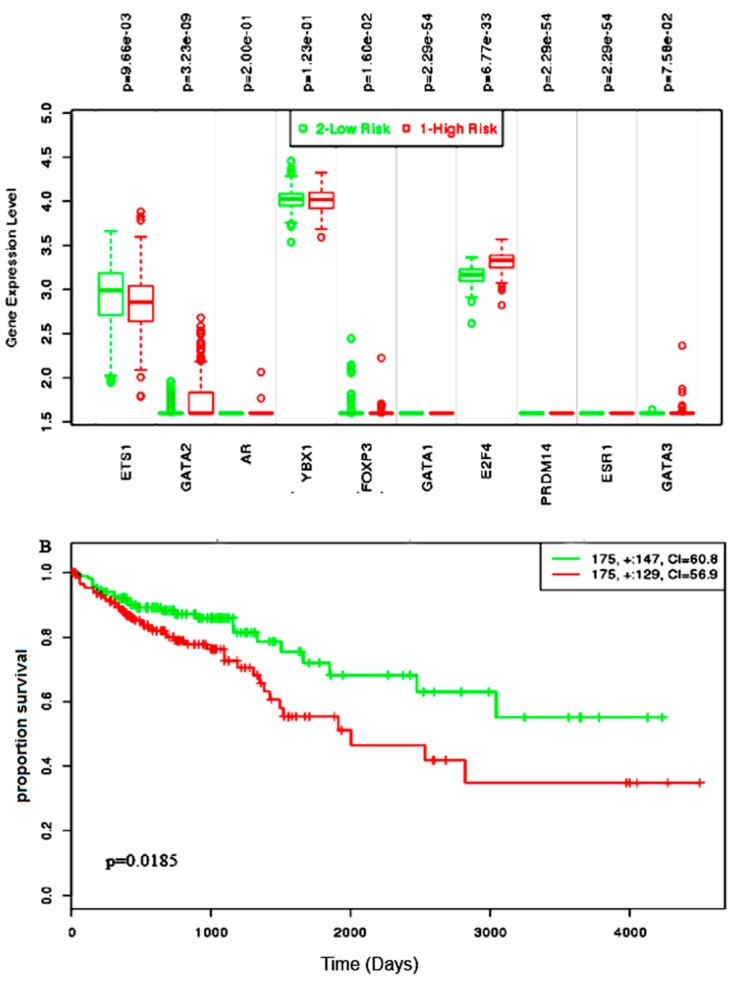
The survival assessment of the reporter transcription factor (TF) signatures in the prognosis of colorectal cancer. (**A)** The box-plot represents the differential expression of the 10 TFs between both risk groups. (**B**) The Kaplan–Meier plot represents the prognostic power of the TF signatures in colorectal cancer.

**Figure 6:**
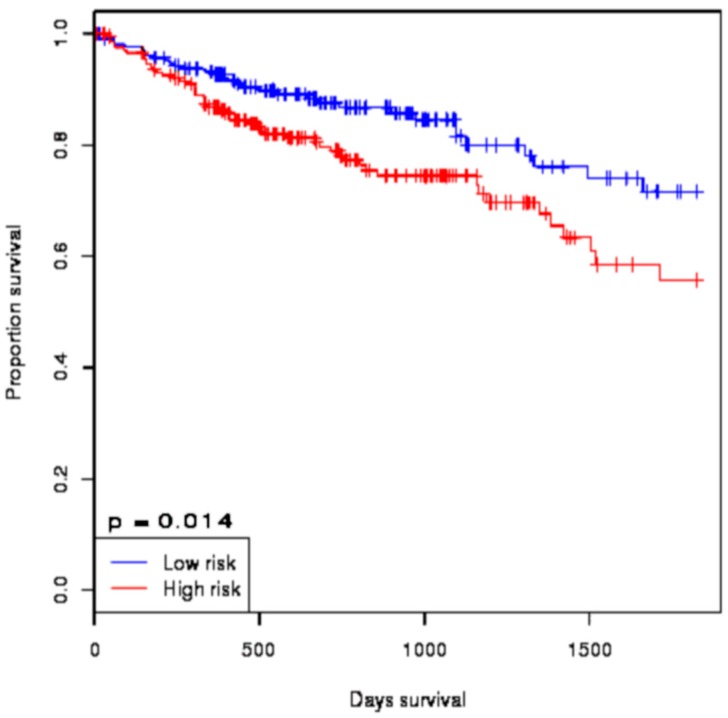
The survival analysis of the reporter microRNA (miRNA) signatures in colorectal cancer. The Kaplan–Meier plot represents the prognostic ability of miRNA signatures (miR-193b-3p and miR-615-3p) in colorectal cancer.

Almost all of the hub proteins (except RFC2) contributed to the discrimination of risk groups as seen in statistical powers represented in the box-plot (Figure 4A), and the hub proteins as a group demonstrated statistically significant prognostic capability with a hazards ratio of 2.57 (log-rank *p* = 9.56 × 10^−6^) (Figure 4B). The reporter TFs (log-rank *p* = 0.0185) were also indicative of CRC prognosis with a hazards ratio of 1.75 (Figure 5B). Among the TFs, GATA1, GATA2, E2F4, ESR1, and PRDM14 were the major discriminators (Figure 5A). In addition, the survival analysis of a subset of reporter miRNAs, consisting of miR-193b-3p and miR-615-3p, showed a prognostic signature (log-rank *p* = 0.014) (Figure 6).

### 3.5. Identification of Candidate Drugs through In Silico Drug Repositioning

**Figure 7:**
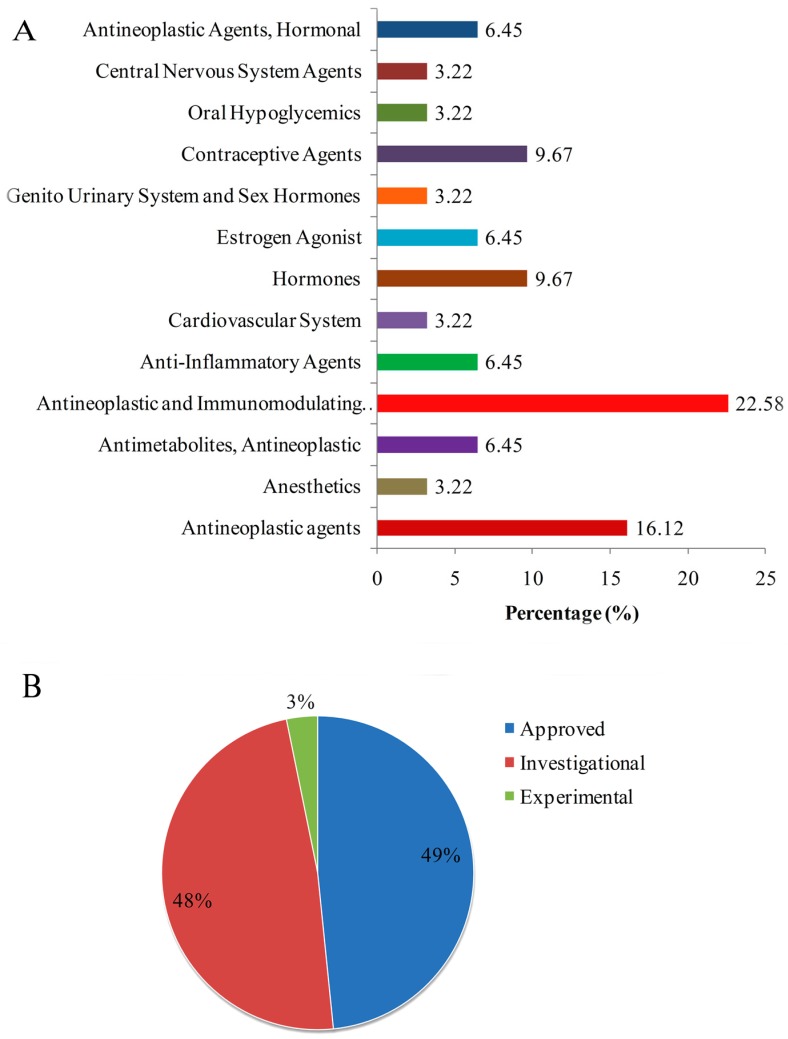
Drug repositioning results in colorectal cancer. (**A**) Classification of repurposed drugs according to drug development stages. (**B**) Distribution of approved drugs into anatomical therapeutic chemical drug classes.

Regarding the hub proteins and TFs as potential drug targets in CRC, we identified potential drugs based on the transcriptome signatures guided drug repositioning tool, geneXpharma, and the CMap database. We considered only the common drugs between both databases for CRC. Statistical evaluation revealed 45 candidate drugs targeting six proteins (Table 4). The drugs were classified according to the anatomical sites and development stages (Figure 7). Among the 10 hub proteins considered as a drug target, three hub proteins, i.e., CCND1, CDK4, and MYC, were targeted by nine drugs (Table 4). Contrarily, among the 10 reporters TFs, three reporter TFs were targeted by 23 drugs (Table 4). The repositioned drugs were classified based on the Anatomical Therapeutic Chemical classification system and it was found that 16.12% were antineoplastic, and 22.58% were antineoplastic and immunomodulating agents. The hormones and contraceptives agents (9.67%) followed the antineoplastic and immunomodulating agents. The repositioned drugs were analyzed and it was found that 49% of drugs were approved, whereas 48% were still under investigation and 3% were in the experimental stage (Figure 7).

## 4. Discussion

Colorectal cancer (CRC) is a complex disease, and the molecular mechanisms of CRC pathogenesis are only partially understood. The augmenting effect of genetic, endocrinological perturbations, and epigenetic aberrations contribute to the pathobiology of CRC [4,5,6]. High-throughput gene expression profiling technology is considered as one of the efficient sources for screening of biomarker candidates [7,8,9]. Understanding the disease pathways and exploration of biomarkers requires integration of omics data from different levels, and the power of this multi-omics approach in the elucidation of molecular signatures in human diseases was previously shown in many human diseases [14,15,16,17,18,19,20,21,22]. Consequently, we employed a systems biomedicine approach to explore the in-depth mechanism of CRC in the present study.

Analysis of differential gene expression in CRC using two different high-throughput experimentations resulted in the identification of 727 upregulated and 99 downregulated DEGs. The pathway enrichment analyses revealed significant molecular pathways including Wnt signaling pathway and inflammatory signaling pathways, which were already implicated in the pathogenesis of CRC [43]. The TGF-β pathway behaves as a tumor suppressor or tumor promoter depending on context in different cancers, and TGF-β was proposed as a target for cancer therapy [44]. Considering the significant alterations in these pathways during the progression of the CRC, we propose their components of as potential therapeutic targets in CRC.

Analysis of the PPI provides insight into central mechanisms on the pathobiology of cancers [45]. The PPI networks were reconstructed in order to clarify the interaction among the identified DEGs. Several hub proteins came into prominence as the reporter signaling mediators in CRC associated PPI. The prognostic survival analysis showed that these hub genes were significantly associated with the worse survival outcomes in CRC patients (Figure 5). Among the hub proteins, ADNP is dysregulated in CRC with high Wnt activity [46]; CEBPB is afflicted with colorectal cancer and glioblastoma cells [47,48]; CCND1 dysregulation contributes to the pathogenesis of CRC [49,50]; CD44 plays diverse roles in cancer cells [51]; CDK4 is the target for different cancer treatments including colorectal cancer [51,52]; CENPA is associated in the pathobiology of CRC [53]; CENPH is also implicated in CRC [54]; RFC2 is implicated in hematologic cancers [55,56]; MYC is dysregulated in CRC [57,58,59]; CENPN is a protein that, in humans, is involved in tge cell-cycle process showing direct binding of CENPN to CENPA [60]. The modules significantly contained the nodes (i.e., CENPA, CENPN, and CENPH) which are associated with different cancers and disease progression as discussed above.

Significant TFs regulating the DEGs were also characterized. Among the reporter TFs, AR is dysregulated in the prostate cancer [61]; ETS is involved in different types of cancers [62]; GATA2 is deregulated in CRC with poor survival outcomes [63]; GATA3 and GATA4 were proposed to be implicated in different cancers [64]; YBX1 and FOXP3 are markers of cancers [65,66,67]; E2F4 disruption is involved in cancers [68,69]; the dysregulation of PRDM14 and ESR1 is found in breast cancers [70,71,72].

The expression of 500 miRNAs was determined in CRC [6]. Thus, we evaluated the biomarker potentiality of the miRNAs in CRC since they regulate genes involved in the cell cycle [12,73,74]. We identified relevant miRNA signatures (miR-193b-3p and miR-615-3p), and survival analysis showed their significant potential as biomarkers in CRC. Recently, Wu et al. found that dysregulation of miR-193b-3p affects the growth of CRC via TGF-β and regulation of the SMAD signaling pathway [75]. Our pathway enrichment results also showed the dysregulation of the TGF-β signaling pathway. Moreover, miR-193b-3p is a predictive biomarker of renal cell carcinoma [76]. The high expression of miR-615-3p is associated with the pathogenesis of CRC and gastric cancer [77,78]. Researches on these miRNAs might provide a therapeutic target for CRC.

The survival analysis of the hub genes, TFs, and miRNAs clarified that these gene signatures (MYC, CENPN, RFC, CENPA, CEBPB, ADNP, CDK4, CCND1, CENPH, and CD44) have high potentiality of being prognostic biomarkers in CRC. It was found that the high expression of reporter TF signatures (AR, GATA1, GATA2, GATA3, EST1, YBX1, PRADM14, ESR1, E2F4, and FOXP3) is associated with worse survival outcomes of the CRC patients. The survival analysis of the miRNA signatures (miR-193b-3p and miR-615-3p) also showed significant prognostic power in CRC. In addition, we here identified 45 candidate repositioned drugs, which were mostly antineoplastics, antidiabetics, and endocrinologicals. 

Despite the tremendous significance of the computational finding of this present work, further experiments at the transcription and protein expression levels (such as Western blot, qRT–PCR, CRISPR/Cas9 gene editing, etc.) and in vitro and in vivo cell culture assays for potential drugs should be performed for confirmation of the above results.

## 5. Conclusions

We employed a well-established systems biomedicine framework where transcriptome datasets were incorporated with genome-scale human molecular networks to reveal molecular biomarker signatures at the RNA (i.e., mRNAs and miRNAs) and protein (i.e., hub proteins and TFs) levels in CRC. The prognostic survival analysis of the identified reporter biomolecules revealed proteomic signatures consisting of hub proteins (MYC, CENPN, RFC, CENPA, CEBPB, ADNP, CDK4, CCND1, CENPH, and CD44), and regulatory signatures consisting of TFs (AR, GATA1, GATA2, GATA3, EST1, YBX1, PRADM14, ESR1, E2F4, and FOXP3) and miRNAs (miR-193b-3p and miR-615-3p) as prognostic biomarker candidates in CRC. In addition, candidate repositioned drugs targeting hub proteins and TFs were identified. The identified biomarker signatures and candidate repositioned drugs in this study deserve further experimentation, since they show importance as candidate biomarkers and therapeutics for precision medicine approaches to treat CRC.

## Figures and Tables

**Table 1:** Functional overrepresentation of differentially expressed genes in colorectal cancer (CRC).

Gene Ontology	Gene Ontology (GO) Term	# of Genes	Coverage (%)	*p*-Value
Upregulated genes				
Biological Process	Collagen fibril organization	11	1.62	4.53 × 10^−7^
	Extracellular matrix organization	22	3.24	2.94 × 10^−6^
	Male gonad development	14	2.06	1.53 × 10^−5^
	Positive regulation of transcription from RNA polymerase II promoter	58	8.56	3.90 × 10^−5^
	Collagen catabolic process	11	1.62	5.07 × 10^−5^
Cellular Component	Extracellular region	84	12.4	2.40 × 10^−5^
	Cytoplasm	216	31.9	5.80 × 10^−5^
	Extracellular space	70	10.3	1.50 × 10^−4^
	Basement membrane	11	1.62	2.56 × 10^−4^
	Extracellular matrix	23	3.39	3.34 × 10^−4^
Molecular Function	Protein binding	354	52.3	8.10 × 10^−8^
	Protein homodimerization activity	42	6.20	7.54 × 10^−4^
	Growth factor activity	15	2.21	1.04 × 10^−3^
	Extracellular matrix binding	6	0.88	1.47 × 10^−3^
	Amino-acid transmembrane transporter activity	7	1.03	4.43 × 10^−3^
Downregulated genes				
Biological Process	Bicarbonate transport	5	4.90	5.89 × 10^−5^
	One-carbon metabolic process	4	3.92	4.00 × 10^−4^
	Chloride transmembrane transport	5	4.90	1.06 × 10^−3^
	Nervous system development	7	6.86	2.63 × 10^−3^
	Regulation of chloride transport	2	1.96	9.62 × 10^−3^
Cellular Component	Plasma membrane	31	30.4	0.0108
	Extracellular space	14	13.7	0.0135
	Integral component of membrane	36	35.3	0.0163
	Anchored component of membrane	4	3.92	0.0179
	Integral component of plasma membrane	13	12.7	0.0421
Molecular Function	Carbonate dehydratase activity	4	3.92	4.16×10^−5^
	Hormone activity	5	4.90	0.0012
	Zinc ion binding	15	14.7	0.0018
	UDP-galactose:β-*N*-acetylglucosamine β-1,3-galactosyltransferase activity	3	2.94	0.0018
	Chloride channel activity	4	3.92	0.0025

**Table 2:** Summary of hub proteins in colorectal cancer.

Symbol	Description	Feature
Hub proteins		
ADNP	Activity-dependent neuroprotector homeobox	Stimulatory and inhibitory effect on the growth of tumor cells
CEBPB	CCAAT/enhancer-binding protein beta	Involved in immune and inflammatory responses
CCND1	Cyclin D1 (afflicted with cancers colonic adenocarcinomas, myeloma)	Cell-cycle regulatory protein
CD44	CD44 molecule	Required in cell–cell interactions, migration
CDK4	Cyclin-dependent kinase 4	Cyclin D1 activates *CDK4*, which causes proliferation of cellular division.
CENPA	Centromere protein A (afflicted with colorectal cancer)	Central role in the assembly of kinetochore
CENPH	Centromere Protein H (afflicted with colorectal cancer)	Central role in the assembly of kinetochore proteins
RFC2	Replication factor C subunit 2	Encodes activator 1 small subunit family
MYC	Myc proto-oncogene	Regulator gene contributes to formation of many human cancers
CENPN	Centromere protein N	Involved in cell-cycle process

**Table 3:** Summary of reporter regulators in colorectal cancer.

Symbol	Description	Feature
Reporter Transcription Factors		
AR	Androgen receptor	Involved in prostate cancer
GATA1	GATA binding protein 1	Transcriptional activator or repressor
GATA2	GATA binding protein 2 (afflicted with colorectal cancer)	Transcriptional activator
GATA3	GATA binding protein 3	Transcriptional activator
E2F4	E2F transcription factor 4	Controls of cell cycle
ETS1	ETS proto-oncogene 1	Involved in tumorigenesis
YBX1	Y-box binding protein 1	Aberrant expression is associated with cancer
PRADM14	PR/SET domain 14	Involved in breast cancer
ESR1	Estrogen receptor 1	Involved in breast cancer
FOXP3	Forkhead box P3 (afflicted with colorectal cancer)	DNA binding
Reporter microRNAs		
miR-193b-3p	MicroRNA 193	Afflicted with CRC and epidermal squamous cell carcinoma
miR-615-3p	MicroRNA 615	Afflicted with CRC
miR-16-5p	MicroRNA 16	Potential biomarkers in gastric cancer
miR-26b-5p	MicroRNA 26	Afflicted with CRC
let-7b-5p	MicroRNA 7	Afflicted with CRC
miR-92a-3p	MicroRNA 92	Afflicted with CRC
miR-124-3p	MicroRNA 124	Afflicted with CRC, gastric and breast cancer
miR-484	MicroRNA 484	Afflicted with CRC
miR-192-5p	MicroRNA 192	Afflicted with CRC
miR-93-5p	MicroRNA 93	Afflicted with head and neck cancer

**Table 4:** Selected repositioned drugs in colorectal cancer.

Target	Repositioned Drug	Drug Class/Status/Description
Hub protein		
CCND1	Gefitinib	Antineoplastic agent; approved; investigational/used in the treatment of cancer
	Hydrocortisone	Anti-inflammatory agent; approved; used in the treatment of inflammation, allergy, collagen diseases, asthma, and some neoplastic conditions
	Irinotecan	Antineoplastic agent; approved; investigational/used in the treatment of colorectal cancer
	Letrozole	Antineoplastic agent; approved; investigational/introduced for treatment of breast cancer
	Lidocaine	Anesthetic; approved; local anesthetic and used as an antiarrhythmia agent
	Methotrexate	Antimetabolite, antineoplastic; approved; antineoplastic antimetabolite with immunosuppressant properties
	Sirolimus	Antineoplastic and immunomodulating agents; approved; investigational/a potent immunosuppressant which possesses both antifungal and antineoplastic properties
	Tamoxifen	Antineoplastic and immunomodulating agent; approved; for the treatment and prevention of breast cancer
CDK4	Gefitinib	Antineoplastic agent; approved; investigational/used in the treatment of cancer
	Lidocaine	Anesthetic; approved; local anesthetic and used as an antiarrhythmia agent
	Sirolimus	Antineoplastic and immunomodulating agent; approved; investigational/a potent immunosuppressant which possesses both antifungal and antineoplastic properties
MYC	Gefitinib	Antineoplastic agent; approved; investigational/used in the treatment of cancer
	Tamoxifen	Antineoplastic and immunomodulating agent; approved; for the treatment and prevention of breast cancer
	Simvastatin	Cardiovascular system; approved; a lipid-lowering agent
Reporter TFs		
GATA3	Azathioprine	Antineoplastic and immunomodulating agent; approved; immunosuppressive antimetabolite pro-drug
	Daunorubicin	Antineoplastic and immunomodulating agent; approved; used in treatment of leukemia and other neoplasms
	Dexamethasone	Antineoplastic agent; approved, investigational, vet approved; for the treatment of endocrine disorders, rheumatic disorders, collagen diseases, dermatologic diseases
	Doxorubicin	Antineoplastic and immunomodulating agent; approved; investigational/used neoplastic conditions like acute lymphoblastic leukemia
	Mercaptopurine	Antimetabolite antineoplastic agent with immunosuppressant properties; approved; in the treatment of leukemia
	Methotrexate	Antimetabolite, antineoplastic; approved; antineoplastic antimetabolite with immunosuppressant properties
ESR1	Clomifene	Estrogen agonist, antagonist; approved; investigational/used mainly in female infertility due to anovulation to induce ovulation
	Daunorubicin	Antineoplastic and immunomodulating agent; approved; used in treatment of leukemia and other neoplasms
	Dexamethasone	Antineoplastic agent; approved; investigational/for the treatment of endocrine disorders, rheumatic disorders, collagen diseases, dermatologic diseases
	Estriol	Estradiol congener; approved; investigational/used as a test to determine the general health of an unborn fetus
	Estrone	Hormone; approved; used for management of perimenopausal and postmenopausal symptoms
	Etoposide	Antineoplastic agent; approved; used in the treatment of refractory testicular tumors and in patients with small cell lung cancer
	Fulvestrant	Antineoplastic and immunomodulating agent; approved; investigational/a drug treatment of metastatic breast cancer
	Glibenclamide	Oral hypoglycemic; approved; used for the treatment of non-insulin-dependent diabetes mellitus
	Imipramine	Central nervous system agent; approved; antidepressant used for the relief of symptoms of depression
	Letrozole	Antineoplastic agent; approved; investigational/introduced for the treatment of breast cancer
	Megestrol	Antineoplastic and immunomodulating agent; approved; investigational/used in the palliative treatment of breast cancer
	Mifepristone	Abortifacient agent and blood-glucose-lowering agent; approved; investigational/for the medical termination of intrauterine pregnancy; also indicated to control hyperglycemia
	Progesterone	Contraceptive agent; approved, vet approved; progesterone acts on the uterus, the mammary glands, and the brain
	Raloxifene	Estrogen agonist, antagonist; approved; investigational/used to prevent osteoporosis in postmenopausal women
	Tamoxifen	Antineoplastic and immunomodulating agent; approved; for the treatment and prevention of breast cancer
	Testosterone	Androgen and estrogen; approved; investigational/in men, testosterone is produced primarily by the leydig cells of the testes; testerone in women functions to maintain libido and general wellbeing.
AR	Cyproterone	Antineoplastic agent and hormone antagonist; approved; investigational/used in the treatment of hypersexuality in males, as a palliative in prostatic carcinoma
	Flufenamic acid	Antiinflammatory and antirheumatic; experimental; analgesic, anti-inflammatory, and antipyretic properties
	Flutamide	Antineoplastic agent, hormonal; approved; investigational/for the management of metastatic carcinoma of the prostate
	Levonorgestrel	Contraceptive agent; approved; investigational/for the treatment of menopausal and postmenopausal disorders
	Mifepristone	Abortifacient agent and blood-glucose-lowering agent; approved; investigational/for the medical termination of intrauterine pregnancy; also indicated to control hyperglycemia
	Spironolactone	Agent causing hyperkalemia; approved; used primarily to treat low-renin hypertension, hypokalemia, and Conn’s syndrome
	Testosterone	Androgen and estrogen; approved; investigational/in men, testosterone is produced primarily by the interstitial cells of the testes; it functions to maintain libido and general wellbeing in women.
